# AI-based disease severity grading predicts complications in laparoscopic appendectomy

**DOI:** 10.1007/s00464-026-13302-6

**Published:** 2026-08-24

**Authors:** Tal Kardish, Monica Ortenzi, Eran Nizri, Danit Dayan

**Affiliations:** 1 Division of General Surgery, Tel Aviv Medical Center, 6 Weizman St., 6423909 Tel Aviv, Israel; 2 Gray Faculty of Medical and Health Sciences, Tel Aviv University, Tel Aviv, Israel; 3 Department of Clinical and Emergency Surgery, Polytechnic University of Marche, Ancona, Italy

**Keywords:** Artificial intelligence, Computer vision, Laparoscopic appendectomy, Disease severity, Complications

## Abstract

**Background:**

Appendicitis severity underpins contemporary management guidelines, where laparoscopic appendectomy remains gold standard. Preoperative measures poorly predict actual disease severity or complication risk, while operative grading systems such as the American Association for the Surgery of Trauma (AAST) remains largely confined to research settings. Artificial intelligence (AI) may provide practical solutions. We evaluated a previously validated AI-derived surgical video assessment of disease severity for predicting perioperative complications.

**Methods:**

This retrospective study included consecutive surgical videos (6/2022–1/2024) routinely analyzed by the AI platform. AI-derived severity scores were stratified into Low (uncomplicated) and High (complicated) groups. Multivariable analysis identified independent predictors of complications. Operative-AAST served for benchmarking. Model discrimination (AUC), post hoc recalibration plot, and decision curve analysis (DCA) were evaluated.

**Results:**

Of 632 cases, 74.5% were low severity and 25.5% high. The High group had higher complication rates (26.7% vs. 10%; p<0.001), including intraoperative (11.8 vs. 3.2%; *p* < 0.001) and postoperative complications (15.5 vs. 7.5%; *p* = 0.005). AI-derived severity independently predicted complications (OR 2.76, 95% CI 1.62–4.73; *p* < 0.001), even after adjustment for operative-AAST (OR 1.90, 95% CI 1.07–3.36; *p* = 0.028). Discrimination was modest (AUC = 0.63), similar to operative-AAST (AUC = 0.68). Calibration plot showed incremental increase in complication rates across probabilities, with acceptable agreements at extremes and improved alignment at intermediate-risk. DCA showed the model had highest net benefit at intermediate-risk, comparable or higher than operative-AAST.

**Conclusions:**

Automated AI-based surgical video assessment shows promise as a complementary tool for risk-prediction of laparoscopic appendectomy. It offers scalable risk stratification that may be implemented in routine clinical practice. Nevertheless, further study and model refinement are warranted.

**Supplementary Information:**

The online version contains supplementary material available at https://doi.org/10.1007/s00464-026-13302-6.

Acute appendicitis is among the most common surgical emergencies worldwide, with a lifetime risk of 7–8%. Between 1990 and 2019, the estimated global age-standardized prevalence and incidence have increased by approximately 20%, reaching 8.7 and 229.9 per 100,000 population, respectively [1]. This situation imposes a significant burden on healthcare systems, therefore efforts are being made to improve cost-effectiveness and management strategies in all aspects. As such, stratifying disease severity into uncomplicated or complicated forms underpins contemporary management guidelines and clinical trials [2].

Laparoscopic appendectomy remains the gold-standard treatment of acute appendicitis [3–5], with reported complication rates of 10% and mortality rates of 1–5% [6], whereas non-operative management is reserved for highly selected patients with uncomplicated disease [7]. The outcomes of laparoscopic appendectomy remain heterogeneous, largely depending on disease severity and surgical complexity. However, current preoperative measures do not reliably predict the true operative findings and perioperative course. Clinical scoring systems and imaging studies are accurate for diagnosing appendicitis but have limited predictive value in this respect [4], and new composite inflammatory indices provide only indirect and limited estimates of disease severity [8].

Recognizing these limitations, the American Association for the Surgery of Trauma (AAST) introduced an anatomic severity grading system based on imaging and operative findings [9]. Although this grading framework correlates with clinical outcomes [10], its use has largely remained confined to research settings, and assessments rely on subjective interpretation documented in narrative operative reports. Such documentation may be associated with limited reliability and incomplete representation of operative reality [11, 12].

Advances in artificial intelligence (AI), machine learning, and computer vision hold promise for providing valuable, practical solutions. Surgical AI platforms enable automated analysis of captured surgical videos into key moments, including scalable and reproducible classification of disease severity, offering potential applications for surgical decision-making support, postoperative risk stratification, and surgical education [13–16].

We previously [17] validated an AI-based computer vision model for operative disease severity grading against expert surgeon review as reference standard in a cohort of 499 laparoscopic appendectomy videos, showing an accuracy of 76.9–94.4% across severity grades (*κ* = 0.77–0.91, *p* < 0.001) with high inter-surgeon agreement (*κ* = 0.86, *p* < 0.001). Higher AI-derived severity was significantly associated with increased intraoperative complexity (*p* < 0.001). Together, these findings support the feasibility and clinical relevance of surgical video-based AI analysis. However, low incidence of postoperative complications limited prognostic utility assessment.

Accordingly, the present study extends this work by analyzing a larger, sequential cohort to evaluate the association between AI-derived disease severity and perioperative complications following laparoscopic appendectomy. Secondary objectives included evaluation of correlations with other perioperative outcomes. We hypothesized that higher AI-derived disease severity is directly associated with increased perioperative complication risk and less favorable outcomes.

## Materials and methods

### Study design and setting

We conducted a retrospective cohort study of consecutive adult patients (≥ 18 years) who underwent laparoscopic appendectomy for acute appendicitis at a tertiary public university hospital between June 2022 and January 2024. All procedures were routinely recorded and analyzed using an AI-based computer vision platform (Surgical Intelligence Platform, Theator Inc., Palo Alto, CA, USA), which automatically generates disease severity scores from video analysis.

The cohort was derived from the AI platform dataset and was procedure-based rather than diagnosis-based, including only surgically treated patients.

The study was approved by the Institutional Review Board with a waiver of informed consent (0014-22-TLV).

### AI platform

The platform applies proprietary AI-based computer vision algorithms to laparoscopic videos, de-identifies extra-corporeal frames, and analyzes recordings within a secure cloud infrastructure [18, 19]. Key operative moments are detected using a two-stage deep learning sequence, combining a 3D deep convolutional neural network with temporal models (Temporal-ConvNet and Long Short-Term Memory network). The first model processes video frames with a vision transformer network to learn spatial and temporal features and the second model identifies temporal segments of surgical actions that define the different annotations [20, 21]. The algorithm development architecture and the most relevant key annotations are shown in Supplementary Figs. 1–3.

### AI-derived disease severity grading

Operative disease severity grading was automatically generated from frame-level visual analysis using predefined laparoscopic criteria, producing a composite five-level severity score previously validated against expert surgeon review [17]. Severity grades were a priori dichotomized into low (grades 1–2) and high (grades 3–5) categories for translational alignment with established clinical stratification (uncomplicated vs. complicated) [22, 23], applied only in downstream outcome analyses.

### AI-derived critical view of safety (CVS)

CVS achievement, defined as correct exposure of the appendix base before transection [24], was automatically identified when frame-by-frame analysis detected complete freeing of the appendiceal tip, appendix inferolateral retraction, terminal ileum visualization at 6 o’clock, and cecal tinea libera at 3 o’clock (entering the appendix base).

### Data collection

AI-derived data were retrieved from the platform dataset, including disease severity, CVS achievement, time to CVS, operative duration, and intraoperative events (bleeding, hemostatic tool use, spillage, appendiceal tear, inadvertent injury). AI events are not necessarily complications.

Clinical outcomes were retrieved from electronic medical records, including intraoperative outcomes (conversion to open surgery, complications), postoperative outcomes (readmissions, total and major 30-day complications according to the Clavien–Dindo classification [25], general or surgical intensive care unit (ICU) admission, reinterventions, and length of stay). Surgical complications included bleeding, superficial and deep surgical site infection, ileus, bowel obstruction, urinary bladder injury.

Imaging and operative-AAST gradings [10] were retrospectively assigned from radiological and narrative operative reports, respectively, for comparative benchmarking [26]. The study cohort did not include cases that were classified as AAST Grade 0 (normal appendix) on either imaging or operative assessment. Imaging-AAST grading was defined according to radiological findings, with appendiceal diameter > 6 mm as a criterion for Grades 1–5, ranging from mild periappendiceal edema (Grade 1) and severe periappendiceal edema (Grade 2), to severe periappendiceal thickening with free fluid in the right lower quadrant or pelvis (Grade 3), abscess or phlegmon (Grade 4), and free fluid involving more than one abdominal quadrant (Grade 5); a nonvisualized appendix was accepted as an alternative finding in Grades 4–5. Operative-AAST grading was defined according to intraoperative findings: intact appendix with inflammation (Grade 1) or gangrene (Grade 2), and perforated appendix with increasing severity of contamination, including local contamination (Grade 3), periappendiceal phlegmon or abscess (Grade 4), and generalized peritonitis (Grade 5). Imaging-AAST grades were dichotomized into low (Grades 1–2) and high (Grades 3–5), and operative grades were dichotomized into low (Grade 1) and high (Grades 2–5), corresponding clinically to uncomplicated and complicated appendicitis and aligned with the AI-derived low- and high-severity categories. AAST groups were analyzed as secondary, contextual comparators to benchmark AI-derived severity against established grading systems. The alignment of dichotomized definitions across imaging-AAST, operative-AAST, AI-derived severity assessment, and clinical classification of complicated versus non-complicated disease is illustrated in Supplementary Fig. 4.

Baseline data were not used by the AI model, described here only for clinical contextualization.

Missing data, including incomplete surgical videos, missing AI-derived disease severity grades, or incomplete complications data, were excluded prior to analysis (*N* = 68). Exclusions were predefined and statistical analyses were conducted using a complete-case approach.

### Outcome measures

Primary outcome: occurrence of any perioperative complication.

Secondary outcomes: operative duration, CVS achievement, length of stay, and readmissions.

### Statistical analysis

Categorical variables (frequencies and percentages) were compared using chi-square or Fisher’s test. Continuous variables (mean ± standard deviation or median with interquartile range, IQR) were tested for normality distribution using Kolmogorov–Smirnov test and compared using the 2 independent-samples *t* test for normally distributed variables or Mann–Whitney *U* test for skewed variables. 95% confidence intervals (CI) were estimated for median values, where applicable, using bootstrap resampling (× 5000).

Binary logistic regression analysis was performed to identify complication predictors. Three multivariable models were constructed, using operative and imaging-AAST grading systems as comparative benchmarks. The first model evaluated the association between AI-derived severity group and complications after adjustment for covariates. The second model evaluated operative-AAST group using the same adjustment variables. The third combined model included both AI-derived severity group and operative-AAST group to assess their independent predictive contributions. Covariates included age, sex, CVS achievement, C-reactive protein (CRP), white blood cell count (WBC), pain duration (before arrival), time to surgery (from diagnosis confirmation), and imaging-AAST group. Bootstrap (× 5000) resampling and percentile-based 95% CIs were performed. Adjusted odds ratios (ORs) with 95% CI were reported.

Statistical significance was defined as a 2-sided *p* value < 0.05. Statistical analyses were conducted using IBM® SPSS® Statistics version 29.0 and R version 4.5.1 (R Foundation for Statistical Computing, Vienna, Austria).

Discrimination was assessed using the receiver operating characteristic (ROC) analysis with estimation of the area under the curve (AUC). ROC curves of the primary AI model and comparative AAST benchmarks were compared, with AUCs reported alongside 95% CIs. Pairwise comparisons of correlated ROC curves were performed using non-parametric methods, and clinical relevance was evaluated by the observed differences in AUC magnitude.

Calibration was evaluated using a calibration plot comparing predicted probabilities of perioperative complications with observed rates across risk deciles, with a 45° reference line representing perfect calibration. To correct for baseline systematic distortions, a formal logistic recalibration (recalibration in-the-large and slope correction) was performed via secondary binary logistic regression to generate the final adjusted curve. Decision curve analysis (DCA) was performed to assess the clinical utility of the prediction model by estimating net benefit across threshold probabilities ranging from 0 to 0.5 [27]. Net benefit was calculated, accounting for the relative weighting of false-positive and false-negative results at each threshold [28]. The prediction model was compared with default strategies (‘treat all’ and ‘treat none’) and the comparative operative-AAST classification. Clinical usefulness was defined as a higher net benefit than alternative strategies across a clinically relevant range of threshold probabilities.

## Results

A total of 632 consecutive patients who underwent laparoscopic appendectomy for acute appendicitis were included in the analysis. Based on the automated surgical video assessment by the AI platform, 471 procedures (74.5%) were classified as low disease severity (Low group) and 161 procedures (25.5%) as high disease severity (High group).

Intraoperative performance metrics according to AI-derived severity classification are summarized in Table 1. CVS was fully achieved in 82.9% of cases, with no significant difference between the groups (*p* = 0.80). However, the time to achieve CVS was significantly longer in the High group (median 26.2 [17.6–37.2] minutes) compared with the Low group (13.0 [9.0–20.3] minutes; *p* < 0.001). Similarly, the total operative duration was markedly prolonged in the High group (42.4 [32.5–59.9] vs. 24.7 [19.0–35.2] minutes, *p* < 0.001). AI-detected intraoperative events, including hemorrhage-related, spillage, and appendiceal tear, were significantly more frequent in the High group (31.7 vs. 9.8%, *p* < 0.001).

**Table 1:** Intraoperative outcomes according to disease severity classification

	Valid *N* (total)	Disease severity			*p*
		Total (*N* = 632)	Low (*N* = 471)	High (*N* = 161)	
CVS fully achieved, %	609	82.9	83.2	82.1	0.8^a^
Time to CVS, min	609	15.2 (10.0–25.0) 95% CI (14.2–16.3)	13.0 (9.0–20.3)	26.2 (17.6–37.2)	< 0.001^b^
Complications, %	632	5.4	3.2	11.8	< 0.001^a^
Bleeding		0.8	0.2	0.6	0.016^a^
Spillage		2.7	1.9	5.0	0.049^a^
Tear of appendix		3.0	1.5	7.5	< 0.001^a^
Conversion, %	632	0.3	0.2	0.6	0.614^a^

Data are presented as percentage, or median (interquartile range %25–75), as appropriate. The 95% confidence interval (CI) for the median was estimated using bootstrap resampling (5000 samples)

*CVS* critical view of safety

^a^Comparisons were performed using the *χ*2 or Fisher’s exact test

^b^2 independent Mann–Whitney *u* test, as appropriate

Intraoperative complications, as documented in the operative notes, occurred in 5.4% of cases with a significantly higher incidence in the High group (11.8 vs. 3.2%, *p* < 0.001). Iatrogenic appendiceal tear and spillage were the most frequent intraoperative complications in the entire cohort (2.7 and 3%, respectively), followed by bleeding (0.8%). Also, there was one case of minor retroperitoneal violation in the High group and 2 cases of superficial cecum injury in the Low group, without further implications. Conversion was rare (0.3%) and did not differ significantly between the groups.

Postoperative outcomes are summarized in Table 2. Total complications occurred in 9.5% of patients, and were significantly more common among the High group (15.5 vs. 7.5%; *p* = 0.005). Deep surgical site infections were the most frequent postoperative complications in the entire cohort (3.3%), followed by superficial surgical site infections (0.8%), ileus (0.6%), and bleeding (0.3%). For this complications subset, the AI-based approach demonstrated risk stratification comparable to operative-AAST grading, distinguishing low- and high-risk groups with complication rates of 3.4% vs. 8.7% (OR 2.71), compared with 3.2 vs. 8.3% (OR 2.74), respectively. There was one case of urinary bladder injury that was diagnosed on readmission and successfully treated with a temporary urinary catheter. This iatrogenic injury was not included in the predefined output set of the AI model training framework and therefore could not be identified by the model. Major complications were also more frequent in the High group (5.6 vs. 1.5%, *p* = 0.008), with a significantly higher rate of grade III complications (5 vs. 1.1%; *p* = 0.006). ICU admission was needed in 0.5% of the entire cohort due to sepsis with organ failure at presentation. There was one case of death (0.2%) in a nonagenarian patient due to acute coronary syndrome at readmission, who refused treatment. Readmissions occurred in 4.0% of patients, without a statistically significant difference between severity groups (*p* = 0.1). Length of stay was significantly longer in the High group (median 2.8 [1.3–4.5] vs. 1.1 [0.8–1.9] days, *p* < 0.001).

**Table 2:** Postoperative outcomes according to disease severity classification

	Valid *N* (total)	Disease severity			*p*
		Total (*N* = 632)	Low (*N* = 471)	High (*N* = 161)	
Readmissions, %	632	4	3.2	6.2	0.102^a^
Total complications, %	632	9.5	7.5	15.5	0.005^a^
Major complications, %	632	2.5	1.5	5.6	0.008^a^
Grade III		2.1	1.1	5	0.006^a^
Grade IV		0.5	0.2	1.2	0.161^a^
Grade V		0.2	0.2	0	1^a^
Length of stay, d	632	1.3 (0.8–2.7) 95% CI (1.15–1.37)	1.1 (0.8–1.9)	2.8 (1.3–4.5)	< 0.001^b^

Data are presented as percentage, or median (interquartile range %25–75), as appropriate. The 95% confidence interval (CI) for the median was estimated using bootstrap resampling (5000 samples)

^a^Comparisons were performed using χ2 or Fisher’s exact test

^b^2 independent Mann–Whitney *u* test, as appropriate

**Fig. 1:**
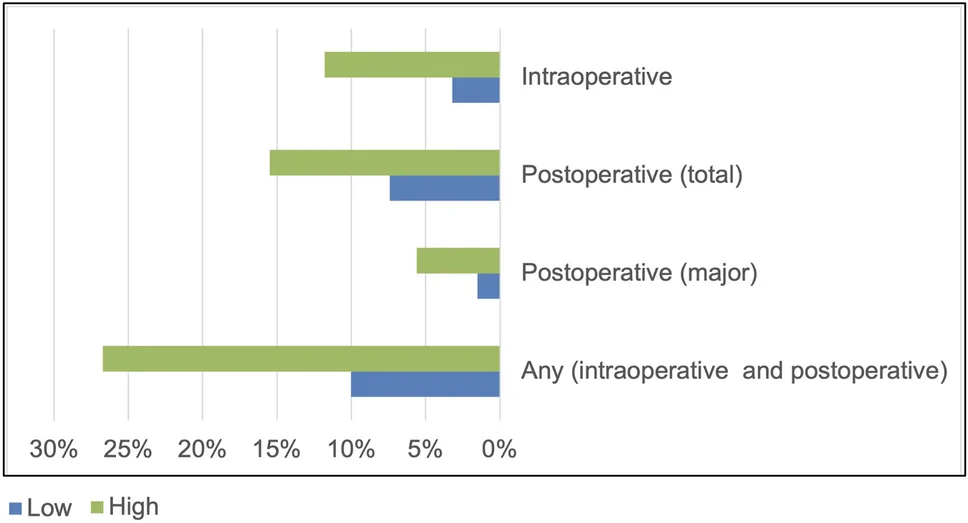
Comparison of surgical complication rates between the high- and low-disease severity groups. Bars represent percentages. Complication rates were significantly higher in the High group across all categories: intraoperative (*p* < 0.001), total postoperative (*p* = 0.005), major postoperative (*p* = 0.008), and any complication (intraoperative plus postoperative, *p* < 0.001)

Surgical complication rates, stratified by disease severity, are shown in Fig. 1.

In multivariable logistic regression analysis, high AI-derived severity group was independently associated with increased odds of any complication (OR 2.76, 95% CI 1.62–4.73, *p* < 0.001). High operative-AAST also demonstrated a strong independent association with complications (OR 4.56, 95% CI 2.51–8.29, *p* < 0.001), whereas imaging-AAST grade was not independently associated with complications after adjustment. In the combined model including both AI-derived severity group and operative-AAST, both variables remained independently associated with complications, although with attenuated effect sizes (AI-derived severity group: OR 1.90, 95% CI 1.07–3.36; *p* = 0.028, and operative-AAST: OR 3.67, 95% CI 1.96–6.86, *p* < 0.001). Model goodness of fit was acceptable (Hosmer–Lemeshow *p* = 0.548), and bootstrap validation demonstrated stable coefficient estimates with minimal deviation between original and resampled models. The combined multivariable binary logistic regression analysis is shown in Table 3. Although the adjusted odds ratio for AI-derived disease severity was lower than that of operative-AAST grade, its confidence interval was comparatively narrower, reflecting good precision of the estimate. Other variables were not significant (*p* > 0.05).

**Table 3:** Multivariable logistic regression predicting any (intraoperative and postoperative) complications

Variable	Adjusted OR	95% CI	*p*
Disease severity group (high)	1.90	1.07–3.36	0.028
CVS fully achieved (yes)	1.45	0.72–2.90	0.299
Imaging-AAST (high)	0.83	0.46–1.51	0.547
Operative-AAST (high)	3.67	1.96–6.86	< 0.001
Sex (male)	0.63	0.38–1.05	0.078
Age (years)	1.00	0.99–1.02	0.652
Pain duration (days)	1.05	0.92–1.21	0.475
Time from diagnosis to surgery (hours)	1.00	0.99–1.03	0.383
CRP (mg/L)	1.00	0.99–1.01	0.247
WBC (× 10⁹/L)	1.03	0.97–1.10	0.365

AAST grading was dichotomized into low (imaging grades 1–2; operative grades 1) and high (imaging grades 3–5; operative grades 2–5), corresponding to uncomplicated and complicated appendicitis, respectively, and aligned with AI-derived low and high disease severity

*AAST* American Association for the Surgery of Trauma, *AI* artificial intelligence, *CRP* C-reactive protein *CVS *critical view of safety, *WBC* white blood cell count

Using operative-AAST grading as a comparative benchmark, the AI-derived disease severity classification demonstrated 77.7% accuracy, 87.7% specificity, 81.5% negative predictive value, 54.9% sensitivity, and positive predictive value 66.3%.

The AI model demonstrated modest discriminative performance, with an AUC of 0.63 (95% CI 0.57–0.70). As a secondary analysis, pairwise comparisons of correlated ROC curves showed that its discriminative ability was comparably modest as imaging-based AAST (AUC 0.57, *p* = 0.24) and operative-AAST benchmarks (AUC 0.68, *p* = 0.23). The observed differences in AUC magnitude between the primary AI model and AAST benchmarks were small and did not meet thresholds considered clinically meaningful. However, cross-stratification analysis (Table 4) highlights its complementary clinical value: the AI model refined risk within operative-AAST low-risk patients, identifying a subgroup with higher complication rates (16.7 vs. 6.8%, *p* = 0.012). Concordant classification further delineated extremes, with the highest complication rates in dual high-risk patients (31.8%) and the lowest in dual low-risk patients (6.8%). Among patients with advanced phenotypes (abscess or localized peritonitis and without significant cecal phlegmon; *N* = 146), 40 postoperative complications occurred. The AI model classified all cases as high severity, whereas operative-AAST under-graded 35.6% of cases (52/146) as low severity. Complications developed in 17.3% (9/52) of these under-graded cases, representing 22.5% of all subgroup complications. This further highlights the clinical value of the AI model in identifying high-risk patients.

**Table 4:** Cross-stratification analysis of perioperative complication rates

O-AAST	AI	Total (*N*)	Complications, *N* (%)	*p*
Low	Low	384	26 (6.8%)	0.012
	High	54	9 (16.7%)	
High	Low	87	21 (24.1%)	0.240
	High	107	34 (31.8%)	

*AI* artificial intelligence, *O-AAST* operative American Association for the Surgery of Trauma (AAST)

**Fig. 2:**
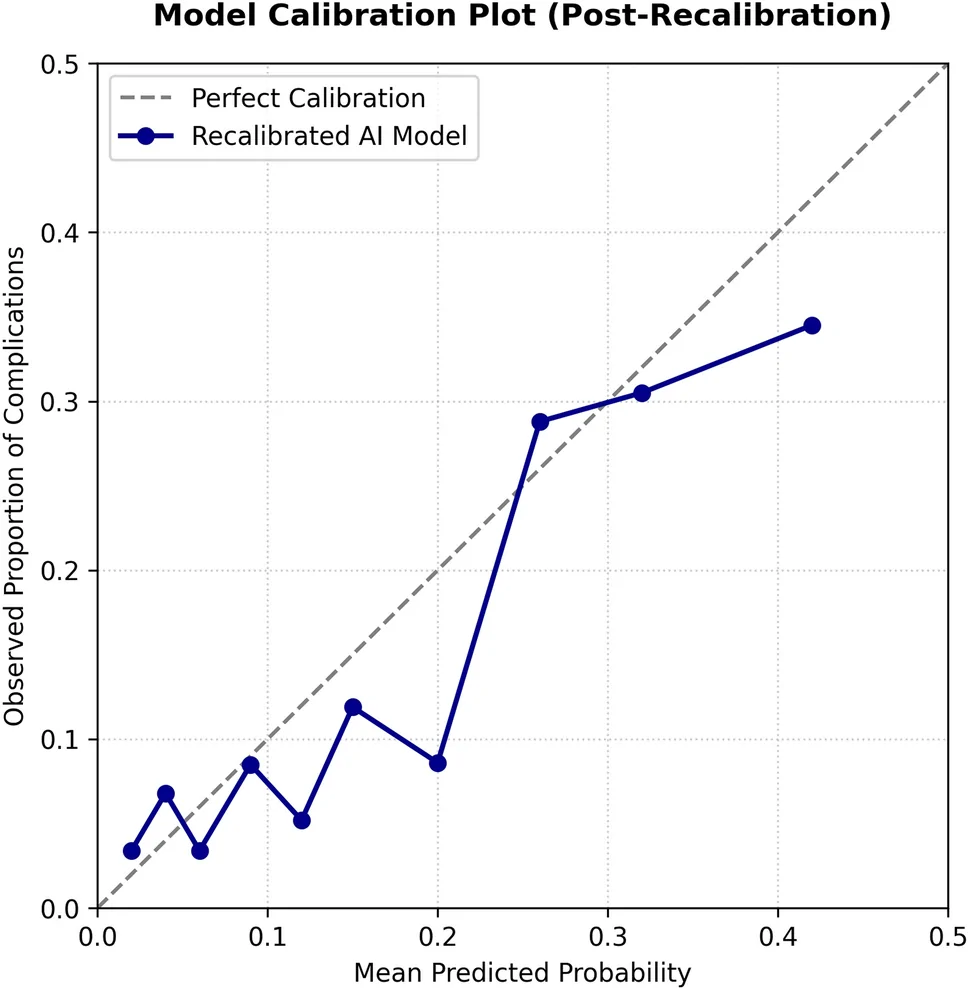
Model calibration plot after post hoc recalibration. The plot demonstrates the relationship between predicted and observed probabilities of complications. The dashed gray line represents ideal calibration. Alignment was acceptable at extremes and improved in intermediate-risk range compared with the original plot

Following post hoc recalibration, calibration plot showed incremental increase in complication rates across probabilities, with acceptable agreements at extremes and improved alignment at intermediate-risk (Fig. 2).

**Fig. 3:**
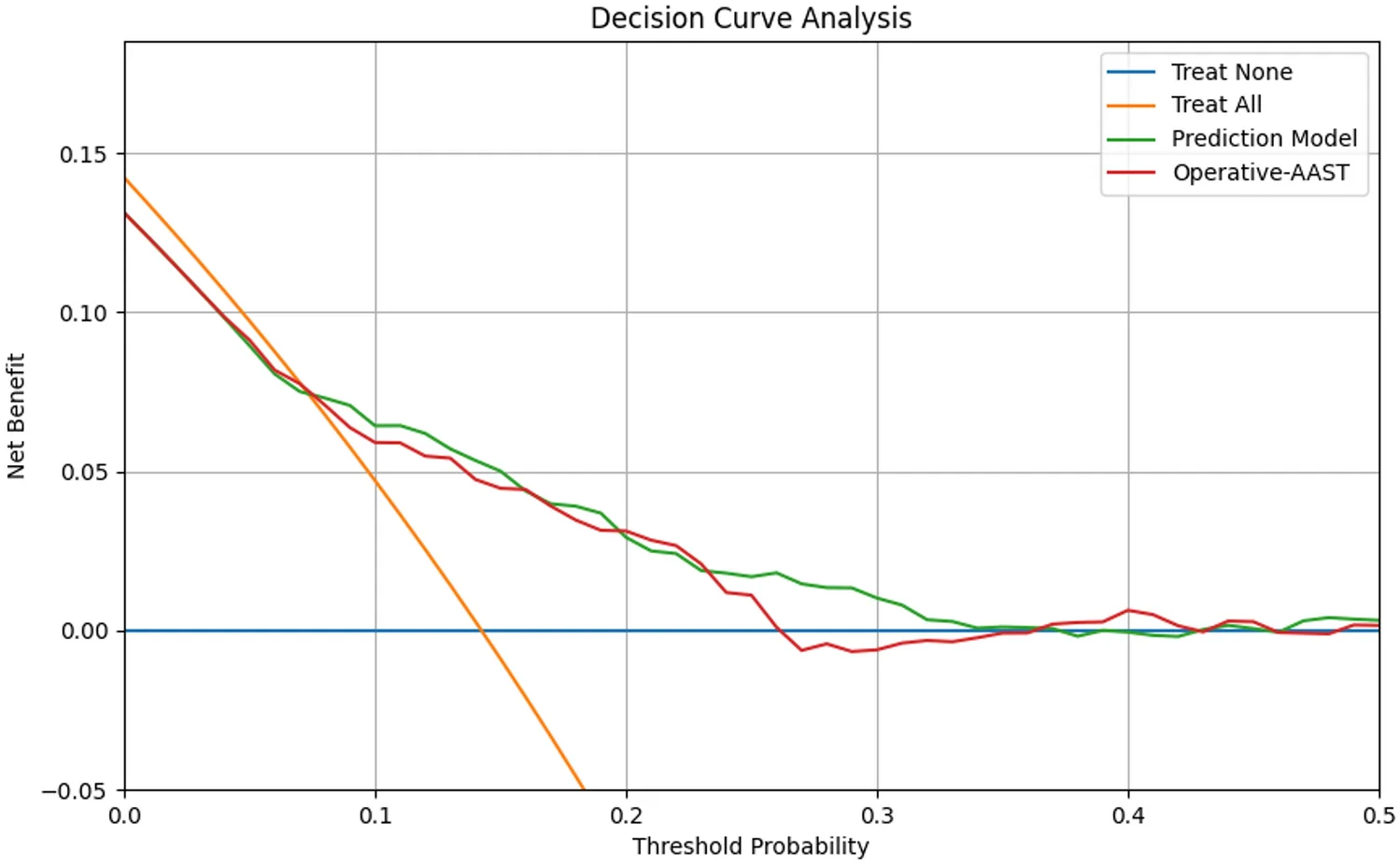
Decision curve analysis for predicting intra- and postoperative complications. The curves illustrate the net benefit associated with the AI-derived prediction model and operative-AAST comparative benchmark, across threshold probabilities of 0–0.50. The x-axis represents the threshold probability, and the y-axis represents net benefit. The ‘treat all’ and ‘treat none’ strategies are shown for comparison. At intermediate-risk range, the AI model showed its highest net benefit, which was comparable or even higher than the comparative benchmark

DCA demonstrated that the prediction model provided higher net benefit compared with both default strategies (‘treat all’ and ‘treat none’) across clinically relevant threshold probabilities (Fig. 3). Net benefit was consistently positive across the evaluated threshold range, indicating that the model provided a favorable balance between correctly identifying patients with complications and avoiding misclassification of patients without complications. The model showed greater net benefit than the ‘treat all’ strategy beginning at approximately 0.07 up to approximately 0.33–0.35, while consistently maintaining superiority over the ‘treat none’ strategy. Compared with the operative-AAST, the prediction model demonstrated comparable or improved net benefit across most thresholds probabilities, with the greatest incremental benefit observed within the intermediate-risk range, suggesting added clinical utility of the AI-derived model in this subset.

Baseline characteristics, used for clinical contextualization, are presented in Table 5.

**Table 5:** Baseline data according to disease severity classification

	Valid *N* (total)	Disease severity group			*p*
		Total (*N* = 632)	Low (*N* = 471)	High (*N* = 161)	
Age, y	632	35.1 (27.3–49.1) 95% CI (33.8–36.0)	33.3 (26.2–44.3)	44.2 (31.4–64.7)	< 0.001^a^
Sex-male, %	632	54.3	53.9	55.3	0.78^b^
Pain duration, d	615	1 (1–2) 95% CI (1–1)	1 (1–2)	2 (1–3)	< 0.001^a^
C-reactive protein, mg/l	623	19.5 (6.3–61.5) 95% CI (17.2–21.9)	15.6 (5.2–39.2)	53.7 (11.0–114.4)	< 0.001^a^
White blood cell count, 10^3^/µl	627	12.5 ± 4.1	12.5 ± 4.2	12.7 ± 3.9	0.67^c^
Neutrophils, %	626	76.1 ± 11.8	75.4 ± 11.3	78.0 ± 12.9	0.02^c^
Appendix diameter, mm	569	11.7 ± 4.0	11.4 ± 4.0	12.7 ± 3.7	< 0.001^c^
Diagnosis to operation time, h	619	11 (6–19) 95% CI (10–12)	11 (6–19)	10.5 (6–18.0)	0.95^a^

The variables shown are provided solely for contextual characterization of the cohort. Data are presented as percentage, mean ± standard deviation, or median (interquartile range %25–75), as appropriate. The 95% confidence interval (CI) for the median was estimated using bootstrap resampling (5000 samples)

^a^2 independent Mann–Whitney *u* test, as appropriate

^b^*χ*2 test

^c^Comparisons were performed using the 2 independent *t* test

## Discussion

In this study, the AI-based operative video analysis platform generated a disease severity metric that independently predicted complications following laparoscopic appendectomy. This suggests that operative video-derived features capture clinically relevant aspects of disease burden not fully reflected by preoperative assessment, translating visual information into a structured metric that directly reflects technical difficulty, risk of iatrogenic injury, and postoperative recovery trajectory [17, 18]. Importantly, this association remained significant even after adjustment for operative-AAST grading, supporting independent contribution to risk stratification.

Despite modest discrimination and reduced intermediate-risk calibration, the model demonstrated complementary clinical utility with favorable net benefit relative to operative-AAST. The model’s superior net benefit over default strategies, namely, the ‘treat all’ and ‘treat none,’ spans a threshold window that corresponds to common clinical decision-making boundaries. This range encompasses the risk spectrum where objective criteria for patient triage are often limited, thereby providing standardized decision support for distinguishing between patients requiring intensive postoperative surveillance and those suitable for standard ward care. It refines risk within conventionally classified low-risk patients and identifies subgroups with higher complication rates that may be under-recognized by standard grading. In complex phenotypes, it consistently classified cases as high severity and flagged patients at increased risk. Collectively, these findings support the role of the AI-based model as an adjunct to existing frameworks, enhancing risk stratification and informing more tailored clinical decision-making, particularly in cases of uncertainty, where a human-in-the-loop approach remains essential.

From a practical standpoint, this performance profile aligns well with clinical needs, as the majority of patients undergoing appendectomy present with lower-severity disease. Operative video-based assessment may facilitate consistent identification of patients suitable for enhanced recovery after surgery (ERAS) pathways, a strategy that reduces length of stay and costs without increasing complications [29–31]. In teaching environments, where laparoscopic appendectomy is commonly performed by residents with variable supervision levels, this could strengthen confidence in management strategy at both resident and team levels.

A key contribution of this AI-based approach lies in bridging the gap from research‑only use of operative grading systems into routine integration in clinical practice for outcome prediction. This is enabled by several elements. First, reliance on objective, video-documented features rather than surgeon perception, supports standardized and reproducible severity stratification, while reducing interobserver variability that is influenced by surgeon experience, case volume, burnout, and cognitive bias [11, 32, 33]. Even when operative findings are comprehensively documented through detailed operative notes or representative intraoperative photographs, their interpretation and severity grading remain dependent on manual assessment by the surgeon or subsequent reviewers. AI-based video analysis may complement such documentation by providing standardized, reproducible, and automated assessment of operative severity. This may facilitate consistent application of established severity classifications and enable their integration into outcome prediction and quality improvement workflows. Second, there is no additional workload or workflow modification. Third, the platform supports extension to large surgical datasets, as continuous video acquisition combined with automated computer vision analysis enables transformation of unstructured intraoperative footage into structured, analyzable data across thousands of procedures [34], reinforcing scalability and robustness. At a health-systems level, this may facilitate cross-institutional comparability, support multicenter quality initiatives, and enable more uniform implementation of ERAS pathways, where uptake remains variable despite guideline endorsement [35, 36].

Similarly, the AI-based operative video analysis approach has demonstrated clinical applicability in laparoscopic cholecystectomy, where AI-derived assessment of disease severity was independently associated with perioperative complications and improved patient risk stratification beyond conventional clinical variables (Hoffman et al. submitted). Likewise, in minimally invasive colorectal surgery, automated AI analysis of indocyanine green fluorescence angiography has been proposed to objectively quantify bowel perfusion and potentially enhance prediction of anastomotic complications [37]. Together, these applications highlight the capacity of computer vision to transform qualitative operative findings into reproducible, objective metrics that could support postoperative risk assessment, clinical decision-making, and surgical quality improvement across diverse procedures.

Nevertheless, implementation and maintenance costs should be evaluated alongside clinical validity to inform broader adoption of such platforms. Comprehensive cost-effectiveness analyses in resource-constrained healthcare settings remain limited [38]. In our institution, platform deployment required dedicated hardware integration with laparoscopic towers, while video storage and analytic processing were performed through a secure cloud-based infrastructure that was readily incorporated into existing operating room workflows at acceptable costs. As the platform supports multiple procedures across surgical subspecialties, fixed implementation costs may potentially be distributed across high procedural volumes, thereby improving cost-efficiency. However, this was not formally examined yet.

Finally, the consistency of disease severity distributions with prior validation work [17], together with the larger cohort and broader outcome spectrum, supports the stability of model performance across successive patient populations. Although temporal variability in resident participation may have influenced outcomes, this reflects routine practice in academic centers, and may therefore enhance, rather than limit, the generalizability of the findings. Overall, these results align with emerging evidence that AI-based computer vision approaches offer incremental value beyond traditional preoperative risk models by quantifying intraoperative disease characteristics that directly influence technical difficulty, complication risk, and recovery trajectory [39–41].

Prior AI models in appendicitis, focused on the diagnosis, demonstrated improved accuracy and efficiency compared with clinical scoring systems [42]. Prospects for the evolution of AI in surgery include machine-learning models with multimodal integration, representing the next critical step. Models capable of combining preoperative clinical and imaging information with operative video-derived features and early postoperative biomarkers (such as serial CRP measurements), are expected to form more accurate and expanded predictive capability to support a dynamic personalized perioperative management strategy.

This study has several limitations. First, its retrospective design introduces the potential for selection bias; however, this was mitigated by including all eligible consecutive cases. Second, the study was conducted at a single academic center where laparoscopic appendectomy is performed by residents without direct supervision. Although this setting may influence generalizability, surgical outcomes were comparable with the reported literature. Third, unmeasured factors may have influenced outcomes, including heterogeneity in resident involvement across procedures. This variability reflects differences in case complexity allocation, level of training, and operative role within the surgical team, as well as hands-on participation. Such heterogeneity precluded robust adjustment for trainee-related effects and limited the ability to assess potential interactions between disease severity and operator experience, including structured escalation pathways (e.g., involvement of a more experienced resident or, when necessary, the attending surgeon), which may have introduced bias into the outcomes. However, this reflects routine practice in academic centers and supports the real-world applicability of the findings. Other unmeasured factors, including patient characteristics (such as frailty) and specific surgical strategies (e.g., stapler use), may have influenced outcomes. However, our cohort reflected the typical appendectomy population, predominantly comprising young and healthy patients, and surgical management was largely standardized, with endoloop ligation used in the vast majority of cases. Future studies should assess model generalizability across diverse patient populations, surgeon experience levels, and variations in surgical techniques. Fourth, the underlying algorithms are proprietary and not transparent, a recognized limitation of many commercially developed clinical AI platforms. However, the model was trained on diverse external datasets, supporting generalizability beyond the study cohort. The platform operates on standard laparoscopic video inputs and produces reproducible outputs, enabling independent validation across different datasets and institutions, even without access to the underlying code, as previously demonstrated [17]. In addition, implementation across multiple institutions and countries supports the feasibility of multicenter evaluation in varied clinical settings. Accordingly, prospective multicenter studies are warranted to further assess external validity, confirm generalizability, evaluate performance stability over time, and determine the clinical impact of AI-based operative video analysis.

In conclusion, this study demonstrates that a video-derived AI metric provides a standardized and reproducible assessment of disease severity and functions as an independent predictor of perioperative complications following laparoscopic appendectomy. This approach adds an objective perspective that complements existing grading systems and leverages risk assessment from research settings into routine clinical practice. By addressing the critical gap between preoperative risk estimation and postoperative outcomes, this approach establishes a foundation for future multimodal predictive systems integrating preoperative, intraoperative, and postoperative data to support more personalized, safe, and efficient surgical care. Prospective, multicenter studies are needed to confirm generalizability and define its optimal integration into routine perioperative care pathways.

## Supplementary Information

Below is the link to the electronic supplementary material.

Supplementary file1 (TIFF 5443 KB)

Supplementary file2 (TIFF 13002 KB)

Supplementary file3 (TIFF 14038 KB)

Supplementary file4 (TIFF 20252 KB)

Supplementary file5 (DOCX 14 KB)

## Data Availability

All data of this study are available within the article.
