# Supplementary material for: AI-based disease severity grading predicts complications in laparoscopic appendectomy
Source: Surg Endosc. 2026 Aug 24;40(9):8139–49. doi: 10.1007/s00464-026-13302-6 (PMC13582077; doi:10.1007/s00464-026-13302-6)
Supplement: Supplementary file 1 [file 464_2026_13302_MOESM5_ESM.docx]

**Supplementary Figure 1.** Algorithm development architecture illustrating the deep feature extraction model and the temporal model. CLS - classification, FV – feature vector, P - positional embedding, PE - positional embedding, ViT - vision transformer.

**Supplementary Figure 2.** Artificial Intelligence-derived annotations of procedural steps (left) and corresponding durations (right).

**Supplementary Figure 3.** Artificial intelligence-derived annotation of “extreme complexity” (i.e., Grade 5 disease severity) in a case of perforated appendicitis with generalized peritonitis (left). The appendiceal critical view of safety (CVS), with annotations indicating the achieved criteria, is shown on the right.

**Supplementary Figure 4.** Alignment of severity classifications illustrating the corresponding dichotomized categories used for comparative analyses across imaging AAST, operative AAST, AI-derived severity assessment, and clinical classification. AAST, American Association for the Surgery of Trauma; AI, artificial intelligence.
